# Consumer awareness and knowledge regarding use of non-steroidal anti-inflammatory drugs (NSAIDs) in a metropolitan area 

**DOI:** 10.3389/fphar.2024.1362632

**Published:** 2024-06-20

**Authors:** Paolo Montuori, Seyedeh Zahra Shojaeian, Francesca Pennino, Daniela D’Angelo, Michele Sorrentino, Salvatore Di Sarno, Raffaele Nubi, Alfonso Nardo, Maria Triassi

**Affiliations:** Department of Public Health, “Federico II” University, Naples, Italy

**Keywords:** non-steroidal anti-inflammatory drugs, knowledge, attitudes, behaviors, multiple linear regression analysis)

## Abstract

**Background:**

Non-steroidal anti-inflammatory drugs (NSAIDs) have well-known adverse effects, and numerous studies have shown inappropriate behaviors regarding their use. The primary aim of this study was to analyze the knowledge, attitudes, and behaviors regarding the use of NSAIDs simultaneously in one of the largest and most populated areas of Italy, Naples.

**Methods:**

From 2021 December 14th to 2022 January 4th, a cross-sectional survey study was conducted among community centers, working places, and universities using a snowball sampling method. For inclusion in the study, the participants were required to be at least 18 years old and residents in the metropolitan area of Naples. Three multiple linear regression analysis (MLRA) models were developed by including variables that could potentially be associated with the following outcomes of interest: knowledge (Model I), attitudes (Model II), and behavior (Model III) regarding the use of NSAIDs.

**Results:**

Data were acquired from 1,012 questionnaires administered to subjects evenly divided by gender with an average age of 36.8 years and revealed that only 7.9% of the participants self-admittedly did not take NSAIDs, while approximately half the participants (50%) admitted to occasionally using them. The results showed a statistically significant correlation between attitudes regarding the appropriate use of NSAIDs and less knowledge. The regression analyses indicated that behaviors regarding the appropriate use of NSAIDs were statistically significant in younger respondents, non-smokers, and those without children. These interesting results showed that behaviors regarding the appropriate use of NSAIDs were significantly higher among respondents with less knowledge and more positive attitudes.

**Conclusion:**

According to the collected data and statistical analysis results, it is possible to identify factors that can greatly affect inappropriate behaviors regarding the use of NSAIDs and establish targeted prevention programs.

## 1 Introduction

Non-steroidal anti-inflammatory drugs (NSAIDs) are a class of pharmaceuticals approved by both the US Food and Drug Administration (FDA) and European Medicines Agency (EMA) for use as antipyretic, anti-inflammatory, and analgesic agents (Bradford and Kleit, 2015; Kaufman et al., 2018; Farkouh et al., 2022; Trasolini et al., 2022). These effects make NSAIDs useful for treating muscle pain, dysmenorrhea, arthritic conditions, pyrexia, gout, and migraines, in addition to being employed as opioid-sparing agents in certain acute traumas (Jahnavi et al., 2019; Freo et al., 2021; Ghlichloo and Gerriets, 2023; Murphy et al., 2023; Sisignano and Geisslinger, 2023). Owing to their efficacies in reducing pain and inflammation, NSAIDs are among the most popularly used medicines, confirming their position in the World Health Organization’s Model List of Essential Medicines (WHO, 2021). They account for approximately 5%–10% of all medication prescriptions each year (Wongrakpanich et al., 2018), and approximately 30 million people worldwide consume NSAIDs every day (Pradère et al., 2020).

Although it is true that NSAIDs are effective for their intended purposes, potential side effects such as major upper gastrointestinal bleeding, acute renal injury, and cardiovascular outcomes such as myocardial infarction (MI) and stroke have also been reported (Kaufman et al., 2018; Vina et al., 2021; Varga et al., 2020; O’Connor et al., 2019; Davis et al., 2017; Green and Norman, 2016). In July 2015, the FDA issued a warning statement on non-aspirin NSAIDs, emphasizing the increased risk of MI and/or cerebrovascular accidents in patients taking these drugs (Rosen et al., 2015; Wongrakpanich et al., 2018; Shaikh et al., 2021). Complications related to NSAID use are responsible for more than 100,000 hospitalizations and 16,500 deaths each year in the United States and contribute to about 2 billion US dollars in healthcare costs (van den Bogert et al., 2017). Many studies have demonstrated the increased risk of peptic ulcer bleeding from approximately three to five fold with regular use of NSAIDs (Hawkins and Hanks, 2000; Silvani et al., 2006; Sostres et al., 2013). Other studies have agreed that the collateral effects of NSAIDs are related to several factors, such as the type of drug, dosage, duration of consumption, patient age, comorbidities, and concomitant use of other drugs (McDonald et al., 2002; Phueanpinit et al., 2017; Vina et al., 2021). Despite being widely used, it has been shown that a large percentage of patients are unclear about the common risks associated with taking NSAIDs (van den Bogert et al., 2017).

Some studies have even demonstrated the inappropriate use of NSAIDs (Ingrasciotta et al., 2019) by describing the demographic and clinical characteristics of elderly analgesic users, evidencing that at least half of the study patients with chronic kidney disease were taking NSAIDs when they should have been avoided (Ingrasciotta et al., 2019). This is particularly worrying when considering that NSAIDs are majorly used for self-medication and therefore without control by a doctor for the type and dosage of drug taken (Wiliński et al., 2015; Doomra and Goyal, 2020; Almohammed, 2023). Indeed, there are several categories of available NSAIDS, some of which necessarily require a prescription and others that are available in many countries as over-the-counter (OTC) drugs (Cavagna et al., 2013; Duong et al., 2014; Moore et al., 2014; Nunes et al., 2016). Ibuprofen, which is available both with and without prescription, is the second most used drug after paracetamol in the US as well as the most used anti-inflammatory in both the US and Australia (Mullan et al., 2017). In addition, consumers could take several types of NSAIDs at the same time, which could entail a cumulative risk (Mullan et al., 2017; Lee et al., 2020). When sold without prescriptions, NSAIDs are labeled with product-specific dosage indications, including a maximum recommended daily dose, to minimize the risk of toxicity (Kaufman et al., 2018). Despite these label indications, it has been shown that 15% of adult users in the US exceed the maximum recommended daily dose of one or more NSAIDs (Kaufman et al., 2018). In addition, a retrospective study of 3,050 subjects with chronic pain showed that 97% of the participants consumed NSAIDs for more than 21 consecutive days (Ussai et al., 2015). The effectiveness and ready availability of these drugs are partly responsible for their increased consumption over the past 20 years (Kaufman et al., 2018; Bekele et al., 2020; Yasmin et al., 2022). According to some estimates, analgesics constitute a good percentage of the OTC drug market, particularly 14% in Europe, 16.5% in the US, and 8.5% in Australia (Stosic et al., 2011). A cross-sectional study conducted by researchers at Aga Khan University in Karachi, Pakistan, showed that the prevalence of self-medication was 76% (Zafar et al., 2008). Another study revealed that the most common drugs used for self-medication were analgesics (88.3%) (Bekele et al., 2020). Fosbøl et al. (2009) studied the pattern of consumption of NSAIDs in the Danish population between 1997 and 2005 through the analysis of the national register of prescriptions; this study also reported that females and over the age of 65 years make greater use of NSAIDs (Fosbøl et al., 2009). Davis et al. (2017) reported a similar conclusion upon studying how NSAID usage patterns in the American population have changed over time (Davis et al., 2017).

There are many studies in literature on the patterns of consumption of NSAIDs by the general population (Motola et al., 2004; Zhou et al., 2014; Davis et al., 2017). However, it is important to not only quantify the use and identify any form of misuse of NSAIDs in the population but also establish any associated factors. One of the methods that can identify the determinants of specific behaviors is the knowledge, attitudes, and practices (KAP) survey model (USAID, 2011). Indeed, a literature search has revealed that practices are the result of knowledge, attitudes, or their interactions. Several studies have investigated the relationship between the general public’s knowledge and/or attitudes and use of analgesics, but most of the findings are mainly focused on selected populations, such as health workers (Green and Norman, 2016; Bekele et al., 2020; Shaikh et al., 2021), student athletes (O’Connor et al., 2019), or patients with chronic conditions such as osteoarthritis (Suciu et al., 2020; Vina et al., 2021). To the best of the authors’ knowledge, only Saengcharoen et al. (2016) have simultaneously examined the knowledge, attitudes, and behaviors on the use of NSAIDs in Thailand in a non-specific cohort. The authors evaluated the knowledge of NSAIDs and factors associated with their inappropriate use among the public; however, the recruited participants were residents living in houses located in blocks along the main street in one of the largest cities in the southern part of the country having a low population density (378,002 residents with a population density of approximately 435 persons/km^2^) (Woranuch Saengcharoen and others et al., 2016).

Therefore, the main aim of this study was to investigate NSAID use in the population of a large metropolitan area and its correlation to knowledge and attitude, so as to develop health education and community-based interventions for developing correct behaviors regarding the use of NSAIDs.

## 2 Materials and methods

### 2.1 Setting and samples

From 2021 December 14th to 2022 January 4th, a cross-sectional survey study was conducted among community centers, working places, and universities using a snowball sampling method. For inclusion in the study, participants were required to be at least 18 years old and residents in the metropolitan area of Naples (the third-largest city in Italy after Rome and Milan, having a population of 914,906 within the city’s governmental boundaries as of 2022). Every participant directly received a questionnaire (available upon request from the corresponding author). Throughout the study duration, skilled interviewers administered the questionnaire to participants on weekdays between 10:00 a.m. and 8:00 p.m. This timeframe was chosen to prevent overrepresentation of non-working individuals in the sample. At the beginning of the survey, the interviewers introduced themselves as representatives of the Department of Public Health at the University of Naples “Federico II.” They provided comprehensive details to the participants regarding the purpose and extent of the research, methodology employed, and voluntary nature of participation. Additionally, the participants were assured that all collected information would be treated anonymously and maintained confidentially. The participants were explicitly informed of their right to discontinue participation at any time without the obligation to provide a reason. Prior to proceeding with the interview, verbal informed consent was obtained from each participant; further, no incentives were provided for participation or survey completion.

To create the questionnaire, a mixed group composed of physicians, pharmacists, chemists, and biologists gathered to carefully review the questions. During evaluation, the questions that were considered irrelevant to the study objectives were either removed or replaced. To confirm the selected questions, 10 individuals were selected to verify the understanding of the objective of the questionnaire. The initial part of the questionnaire focused on assessing the participants’ sociodemographic characteristics and other health-related information, including gender, age, marital status, partner’s occupation, number of children, level of education, and occupation. The second part aimed to explore the participants’ knowledge, attitudes, and behaviors regarding the use of NSAIDs through a total of 32 questions. The knowledge and attitude items were evaluated on a 3-point Likert scale through the responses “agree,” “uncertain,” and “disagree,” while the items in the behavior section were assessed using a four-option format of “never,” “sometimes,” “often,” and “yes/always.”

### 2.2 Statistical analysis

IBM SPSS (version 27) statistical software was used to analyze the study that was conducted in two stages. To summarize the basic characteristics of the statistical units, a descriptive statistic was used for the first stage, while multiple linear regression analysis (MLRA) models were used in the second stage; MLRA is a statistical technique that uses multiple independent variables to predict the outcome of a dependent variable. The MLRA aims to obtain the linear associations between the independent and dependent variables. The main results obtained from the MLRA include the statistical significance of the regression model (*p*-value <0.05), estimation and statistical significance of the beta coefficients (*p*-value <0.05), and coefficient of determination (R-squared and adjusted R-squared values). The coefficient of determination indicates the extent to which the variations can be accounted by the independent variables.

Three MLRA models were developed by including variables that could potentially influence the following outcomes of interest:

1) knowledge about NSAIDs and their related risks (Model I);
2) attitudes toward predisposing factors to the use of anti-inflammatories (Model II);
3) actual behaviors regarding the use of NSAIDs (Model III).

Knowledge, attitudes, and behaviors were considered as the dependent variables, and their outcomes were obtained by adding the scores from their corresponding questions. Questions with inverse answers were inversely coded. Independent variables were also included in each model by taking into account the gender (1 = male, 2 = female), age (in years), educational level (1 = primary school, 2 = middle school, 3 = high school, and 4 = university degree), marital status (1 = single, 2 = in a relationship), profession (?), smoking habit (1 = smoker, 2 = non-smoker), and whether or not they had children (1 = yes, 2 = no). The components of knowledge were added to the independent variables of Model II, and the components of both knowledge and attitudes were added to those of Model III. During the analysis, attitudes and knowledge were considered as indices rather than scales; this meant that each observed variable (A1, . . ., A10 and K1, . . ., K11) had a causal influence on the corresponding latent variables (attitude and knowledge), indicating that the interactions between the two variables were considered formative indicators that did not require a correlation of the interobserved variables. On the contrary, as evidenced by a Cronbach’s alpha of 0.825, the observed variables (B1, . . ., B11) and the latent variable behavior were reflective indicators. The statistical tests were two-tailed in nature, and the results were considered to be statistically significant for *p*-values equal to or less than 0.05.

## 3 Results

A total of 1,060 anonymous questionnaires were distributed, of which 1,012 were filled and collected for a response rate of 95.47%. Table 1 summarizes the characteristics of the study population. With regard to gender, the population was balanced and reflected the general distribution; out of the 1,012 participants, 543 (53.7%) were women and 469 (46.3%) were men. The mean age of the sample population was 36.8 years, within a range of 18–89 years, and the most represented age group was that between 18 and 30 years (34.3%). A majority of the participants (698) said that they were not single; this distribution is typically representative of the Italian metropolitan population as well as the European and global ones.

**TABLE 1:** Study population characteristics.

Study population	N	Percentage
Sex	1,012	
Male	469	46.3
Female	543	53.7
Age in years		
18–30	347	34.3
31–35	207	20.5
36–40	186	18.5
41–45	77	7.7
46–50	53	5.3
51–65	109	10.9
>65	33	3.3
Marital status		
Single	314	31
In a relationship	698	69
Education		
Primary school	39	3.85
Middle school	96	9.49
High school	501	49.41
Degree	377	37.25
Having children		
Yes	407	40.21
No	605	59.78
Smoking habit		
Yes	441	43.58
No	571	56.42

Table 2 shows the knowledge of the respondents toward use of NSAIDs. As seen from the results, the overall knowledge of the respondents was not excellent. Indeed, a significant percentage of respondents ignored the chances of potential side effects (>20%), including gastric ulcers (>20%), even if 82.01% of the respondents knew that it was preferable to take these drugs on a full stomach. Of specific interest is the finding that less than half of the participants were aware that NSAIDs could cause addiction (31.72%). Approximately 49.41% of the respondents were aware that 15% of people who use anti-inflammatories tended to exceed the recommended dose. Almost all participants knew that NSAIDs are drugs (96.93%). These results are in line with the findings of several previous studies. Mullan et al. (2017) concluded that ibuprofen users in Australia lacked knowledge about the maximum daily dose, contraindications, and potential side effects (Mullan et al., 2017). A recent study conducted in Saudi Arabia by Almohammed (2023) noted that the knowledge of the general population regarding the harmful adverse effects of NSAIDs needed to be improved (Yasmin et al., 2022). Similarly, Kaufman et al. (2018) conducted a national study in the United States and concluded that most users did not recognize that the products they were taking were NSAIDs and that 11% exceeded the maximum daily dose of ibuprofen (Kaufman et al., 2018). There are strong correlations between the corrected responses to questions regarding the appropriate use of NSAIDs and the younger female population in relationship as well as those with higher levels of education (*p*-value <0.002) (Model I in Table 5).

**TABLE 2:** Knowledge of respondents regarding the use of non-steroidal anti-inflammatory drugs.

N	Statement (variables)	Agree (%)	Uncertain (%)	Disagree (%)
K1	Non-steroidal anti-inflammatories are drugs	96.93	1.98	1.09
K2	Non-steroidal anti-inflammatories have no side effects	10.78	12.15	77.07
K3	The use of non-steroidal anti-inflammatories can lead to gastric ulcer	75.49	22.23	2.28
K4	Non-steroidal anti-inflammatories should preferably be taken on an empty stomach	3.56	11.07	85.37
K5	Non-steroidal anti-inflammatories should preferably be taken on a full stomach	82.01	12.94	5.05
K6	Non-steroidal anti-inflammatories cause addiction	31.72	41.60	26.68
K7	It is not recommended to take different types of non-steroidal anti-inflammatories at the same time	66.00	14.32	19.68
K8	Non-steroidal anti-inflammatories always require a prescription	37.35	28.46	34.19
K9	Non-steroidal anti-inflammatories are always refundable by the National Health Service	13.24	39.23	47.53
K10	15% of people taking non-steroidal anti-inflammatories exceed the recommended dose	49.41	42.29	8.3
K11	Paracetamol is a non-steroidal anti-inflammatory drug	50.30	15.37	34.33

Table 3 shows participant attitudes regarding the use of NSAIDs. The survey answers show that approximately 66.3% of the participants considered buying NSAIDs to be too simple, 33.5% believed that they should always be free, and only half of the sample population believed that these drugs should not be sold without a prescription. Furthermore, most participants believed that NSAIDs should be taken only after reading the instructions on the package (81.22%) and in case of extreme necessity (75.79%); approximately 22.53% of the participants believed that they should be taken when the pain was still mild, and approximately 24.60% of respondent considered that these drugs could prevent the onset of pain. It is interesting to note that 53.16% of respondents considered it important to always have an anti-inflammatory preparation with them. The results of the regression analysis (Model II in Table 5) indicate that attitudes regarding the appropriate use of NSAIDs were significantly higher among single respondents (*p*-value: 0.0002) and people with children (*p*-value = 0.000). Interestingly, data on the statistically significant correlations between attitudes regarding the appropriate use of NSAIDs and a lower level of education (*p*-value = 0.000) as well as knowledge (*p*-value = 0.000) showed that improving attitudes regarding the appropriate use of NSAIDs is necessarily dependent on direct interventions regarding knowledge. The attitudes also lack statistically significant correlations with age (*p*-value = 0.073), gender (*p*-value = 0.504), and smoking habits (*p*-value = 0.212) (Model II in Table 5).

**TABLE 3:** Attitudes of respondents toward use of non-steroidal anti-inflammatory drugs.

N	Statement (variables)	Agree (%)	Uncertain (%)	Disagree (%)
A1	Non-steroidal anti-inflammatories should not be sold without prescriptions	51.18	22.63	26.19
A2	Side effects of non-steroidal anti-inflammatories are advertised properly	16.90	32.61	50.49
A3	Being over-the-counter drugs, non-steroidal anti-inflammatories are very safe drugs	12.54	37.15	50.31
A4	It is important to take a non-steroidal anti-inflammatory when the pain is still mild	22.53	20.95	56.52
A5	It is important to always have an anti-inflammatory drug with you	53.16	16.30	30.54
A6	Non-steroidal anti-inflammatories should always be free	33.50	18.18	48.32
A7	Non-steroidal anti-inflammatories help prevent the onset of pain	24.60	13.83	61.57
A8	Buying non-steroidal anti-inflammatories is too simple	66.30	18.08	15.62
A9	You should not take a non-steroidal anti-inflammatory without having read the package leaflet	81.22	9.98	8.80
A10	Non-steroidal anti-inflammatories should be taken in case of extreme necessity	75.79	18.28	5.93

Table 4 shows participant behaviors regarding the use of NSAIDs. The data indicate that only 7.9% of the participants admitted to not consuming NSAIDs, while half of the participants (50%) admitted to occasionally using them. The majority of participants stated that they were taking NSAIDs correctly, while more than 30% of respondents admitted to taking NSAIDs on an empty stomach, with a similar percentage acknowledging that they did not follow their doctor’s prescriptions or instructions on the package correctly (approximately 25%). Consequently, about half of the participants declared consuming NSAIDs more than three times a month, 20% declared taking them for more than 7 consecutive days, and about one-third of the respondents declared taking higher doses than recommended with the expectation of persistent pain. The results of the regression analysis indicate that behaviors regarding the appropriate use of NSAIDs were statistically significant in younger respondents (*p*-value <0.000), non-smokers (*p*-value <0.044), and surprisingly those without children (*p*-value <0.001) (Model III in Table 5). These interesting results show that behaviors regarding the appropriate use of NSAIDs were significantly higher among respondents with less knowledge (*p*-value <0.000) and more positive attitudes (*p*-value <0.001). No correlations were observed between behaviors regarding the use of NSAIDs and gender (*p*-value = 0.848), marital status (*p*-value = 0.060), as well as education level (*p*-value = 0.639) (Model III in Table 5).

**TABLE 4:** Behaviors of respondents toward use of non-steroidal anti-inflammatory drugs.

N	Questions	Yes (%)	Often (%)	Sometimes (%)	Never (%)
B1	Do you take non-steroidal anti-inflammatories?	36.86	5.24	50.00	7.90
B2	Do you take non-steroidal anti-inflammatories without consulting your doctor?	49.90	10.08	25.30	14.72
B3	Do you suggest to your friends to take non-steroidal anti-inflammatories?	21.64	9.19	39.92	29.25
B4	Do you carry a non-steroidal anti-inflammatory with you?	36.66	13.75	22.43	27.16
B5	Do you take non-steroidal anti-inflammatories more than three times a month?	19.17	7.51	31.92	41.40
B6	Do you take non-steroidal anti-inflammatories for more than 7 consecutive days?	5.04	6.03	8.40	80.53
B7	Do you buy non-steroidal anti-inflammatories sponsored on TV?	9.78	4.45	18.38	67.39
B8	Do you take non-steroidal anti-inflammatories on an empty stomach?	7.41	7.41	15.51	69.66
B9	If the pain does not subside, do you take a higher dose of non-steroidal anti-inflammatory than recommended?	10.47	5.04	12.94	71.55
B10	Do you follow the recommendations on the correct use of non-steroidal anti-inflammatories indicated on the package?	75.59	14.82	1.58	8.00
B11	Do you correctly follow your doctor’s prescriptions regarding non-steroidal anti-inflammatories?	69.37	18.47	6.03	6.13

**TABLE 5:** Results of the multiple linear regression analysis (MLRA) models.

Model I (*p-value: 0.0000*)—dependent variable: knowledge	Estimate	Standard error	*p*-value
Age	−0.0266987	0.00824559	0.0012
Sex	0.512349	0.170556	0.0027
Marital status	1.27054	0.198455	0.0000
Smoking habit	−0.116974	0.170354	0.4923
Having children	0.0871074	0.235817	0.7118
Education	1.47335	0.116848	0.0000
Model II (*p-value*: 0.0000)—Dependent variable: Attitudes			
Knowledge	−0.465583	0.0356178	0.0000
Age	−0.0167475	0.00935892	0.0735
Sex	0.129023	0.193446	0.5048
Marital status	−0.849952	0.228609	0.0002
Smoking habit	−0.240015	0.192399	0.2122
Having children	1.74412	0.26629	0.0000
Education	−0.809058	0.141992	0.0000
Model III (*p-value*: 0.0000)—Dependent variable: Behavior			
Knowledge	−0.407143	0.0665074	0.0000
Attitudes	0.857457	0.0544763	0.0000
Age	−0.074477	0.0161805	0.0000
Sex	0.063681	0.333987	0.8488
Marital status	0.745528	0.397317	0.0606
Smoking habit	−0.666786	0.332364	0.0448
Having children	−1.45959	0.46937	0.0019
Education	0.116541	0.249029	0.6398

## 4 Discussion

The main objective of this study was to identify the specific target population of a metropolitan area toward whom health education programs aimed at appropriate use of NSAIDs should be addressed. Hence, the knowledge, attitudes, and behaviors regarding appropriate use of NSAIDs were surveyed in a large cosmopolitan cohort. The results of this study are particularly interesting and numerous.

It is observed from the results that the knowledge of the respondents was generally not excellent. Indeed, more than 20% of the responders were unaware of the potential side effects related to the use of NSAIDs, including gastric ulcers. These results are in line with the results of other studies that investigated the risk perception of NSAIDs. In particular, Varga et al. (2020) investigated the risk perception of NSAIDs in South Dakota in comparison to previous studies conducted in Slovakia and Greece; they found that over half of the cohort had no knowledge of the possible adverse drug reactions (ADRs) connected with the use of NSAIDs and that awareness of the potential adverse cardiovascular consequences was minimal (Varga et al., 2020). Mullan et al. (2017) highlighted that many consumers of NSAIDs had gaps in their knowledge regarding the safe use of these medications (Mullan et al., 2017). Wiliński et al. (2015) concluded from their survey that the level of awareness among young medical students in Poland regarding the risks associated with NSAID usage was quite low (Wiliński et al., 2015). Given the universal availability of NSAIDs, there could be a probable misconception that these drugs are safe for common use. A cross-sectional study by van den Bogert et al. (2017) in the state of Alabama focused on the risk knowledge related to the use of NSAIDs in long-term users through screening questions, it was concluded that increased advice from doctors and pharmacists on the risks of NSAIDs would be beneficial for patient safety (van den Bogert et al., 2017). However, patient perceptions of the risks of NSAIDs were relatively low and often lacking in knowledge about the common adverse effects (Phueanpinit et al., 2017; Ho et al., 2020). All these studies concluded that there was a lack of knowledge about the appropriate use of NSAIDs, but there was no identification regarding the specific cohorts toward whom health education programs should be addressed to increase knowledge and consequently correct behaviors on the use of NSAIDs. In their study, Barkin et al. (2010) focused on the use of NSAIDs in elderly patients and recognized that adverse events in these patients were often caused by comorbidities and risk of interactions with other drugs; in fact, this study concluded that NSAID prescriptions must be patient-specific, patient-focused, patient-centered, and personalized to the individual patient to ensure greater security in administering NSAIDs to the elderly (Barkin et al., 2010). The findings of this study indicate that people in metropolitan areas have gaps in their knowledge regarding the appropriate use of NSAIDs and that targeted health programs must be implemented to increase awareness; further, such efforts should be specifically targeted at i) male, elderly, single, and less educated people with the aim of improving their knowledge (Table 5, Model I) as well as ii) young women in relationships and having high educational levels to improve their knowledge in terms of the appropriate use of NSAIDs. Indeed, as can be seen from the regression analysis of Model III in Table 5, positive behaviors toward the appropriate use of NSAIDs were significantly higher among respondents with less knowledge (*p*-value <0.001). Therefore, the results showed that participants with better knowledge were those with high levels of education. This indicates that such people are less careful regarding the use of NSAIDs because of their knowledge on the subject. Contrarily, those with less knowledge were more inclined toward better usage practices since they were not aware of the possible risks. These findings are in agreement with those of the study by Bekele et al. (2020), who concluded that self-medication was widely practiced among medical and pharmacy students.

Similar to the findings on knowledge, the attitudes were also not excellent in the present study population. Indeed, the answers showed that almost half of the participants were not properly advised on the side effects of NSAIDs; furthermore, more than half of the participants considered NSAIDs to be very safe drugs. About 51.18% of the respondents believed that these drugs should not be sold without prescription. Significant variabilities have been reported in different studies regarding attitudes toward appropriate use of NSAIDs. Bekele et al. (2020) conducted a study among medical and pharmacy students and found positive attitudes toward the use of NSAIDs as well as statistically significant differences between the two categories (Bekele et al., 2020). Grimmer et al. (2002) examined a cohort of physiotherapists from two different areas (rural and metropolitan) and highlighted the need for further education regarding the use of NSAIDs in clinical practice as well as a range of issues that must be addressed in such education programs(Grimmer et al., 2002). Similarly, Green and Norman (2016) concluded that physiotherapists should access and incorporate up-to-date and comprehensive information on appropriate NSAID use into their practice, particularly the side effects, contraindications, and drug interactions (Green and Norman, 2016). In another recent study, Shaikh et al. (2021) confirmed that most physiotherapists recommended NSAIDs to patients despite their poor understanding of the health risks, adverse reactions, and drug interactions of NSAIDs (Shaikh et al., 2021). Consistent with our results, Saengcharoen et al. (2016) revealed inappropriate attitudes as well as limited knowledge regarding the use of NSAIDs in the Thai population (Saengcharoen et al., 2016). Another study in Thailand by Phueanpinit et al. (2017) indicated that orthopedic doctors had positive attitudes on providing information on the ADRs to patients but that the risk information on NSAIDs needs to be improved (Phueanpinit et al., 2017). Furthermore, as demonstrated by the regression analysis (Model II in Table 5), attitudes regarding the appropriate use of NSAIDs were significantly higher among single respondents (*p*-value: 0.0002), those with children (*p*-value = 0.000), and those with less education (*p*-value = 0.000). No statistically significant correlations were observed between attitudes and age (*p*-value = 0.073), gender (*p*-value = 0.504), or smoking habits (*p*-value = 0.212) (Model II, Table 5). Therefore, these findings indicate that targeted health programs need to be implemented to increase attitudes toward appropriate use of NSAIDs and that these efforts must be specifically directed toward people in relationships without children and high levels of education living in metropolitan areas. These results are consistent with those reported in a recent cross-sectional study by Vina et al. (2021) in 334 adults with knee and/or hip osteoarthritis; these authors analyzed the role of knowledge and attitudes regarding the appropriate use of NSAIDs and concluded that future interventional studies could potentially evaluate the effects of changes in such knowledge and attitudes.

An analysis of the behaviors in Table 4 shows that almost all the participants used NSAIDs (only 7.9% responded with “never”). The results also showed that approximately 90% of the respondents consistently followed the recommendations on the correct use of NSAIDs provided on the package (75.59% “Yes” and 14.82% “Often”), and a slightly similar proportion followed their doctors’ recommendations on its use (69.37% “Yes” and 18.47% “Often” for a total of 87.84%). However, more than 80% of people consume NSAIDs without consulting their physicians, according to a recent study by Bekele et al. (2020) (79.7%) and similar studies conducted in Pakistan, Serbia, and India, where the prevalence of self-medication was reported to be 76%, 79.9%, and 78.6%, respectively (Bekele et al., 2020). These findings highlight an important problem: without consultation of a physician, the use of NSAIDs can result in health risks caused by interactions with other drugs (such as anticoagulants, corticosteroids, and some groups of antihypertensive drugs). Moreover, awareness of the risk factors before prescription is important to minimize the side effects during treatment. A more accurate outcome was reported in a study conducted in Kuwait, where the overall prevalence of self-medication was 97.8%. In the current survey, almost 70% of those interviewed consumed NSAIDs on an empty stomach and about 30% declared using a higher dose than recommended. These results are in line with other previous studies; in fact, in the survey conducted in Thailand by Saengcharoen et al. (2016), it was found that about 62% of respondents used NSAIDs with meals, while the rest ingested them 0.5–1 h after meals, before meals, or at bedtime(Saengcharoen et al., 2016). In 2018, O’Connor et al. conducted a study with Irish student athletes, and the participants reported that they used NSAIDs to treat injuries (75.2%) and block pain (60.4%); however, it was also interesting that 13.9% used more than the daily recommended dosage and commonly took NSAIDs as OTC drugs rather than by prescription (O’Connor et al., 2019). In addition, 12.5% and 6.6% of these students used NSAIDs for longer than 10 and 14 days, respectively. Similar results were obtained by Kaufman et al. (2018) in a study conducted in the US from May 2015 to March 2016, where they found that 15% of adult users of ibuprofen in the US exceeded the maximum recommended daily dose of one or more OTC or prescription NSAIDs during the week that they used ibuprofen and that this occurred when the pain was more severe (Kaufman et al., 2018).

As seen from the regression analysis (Model III, Table 5), behaviors regarding the appropriate use of NSAIDs were statistically significant in younger respondents, non-smokers, and those without children. No correlations were observed between behaviors regarding the use of NSAIDs and gender (*p*-value = 0.848), marital status (*p*-value = 0.060), and education level (*p*-value = 0.639). There are several conflicting results among studies that have found correlations between the appropriate use of NSAIDs and sociodemographic factors, such as age and sex. Davis et al. (2017) examined an analytic dataset of 16,533 individuals from the National Health and Nutrition Examination Survey (NHANES) and observed that women in the youngest age group (≥20 to <40) were more likely to be regular NSAID users than men, while this was not noted for any other age group. Usually, women use NSAIDs more than men because they can be useful for the treatment of menstrual pain in women of reproductive age (Davis et al., 2017). Shaikh et al. (2021) indicated a higher risk of NSAID use in patients with a prior history of ischemic stroke (IS) or transient ischemic attack (TIA), especially in younger patients and men (Shaikh et al., 2021). However, osteoarthritis patients of lower age (Albert et al., 2008; Abbate et al., 2018), of female sex (Dominick et al., 2004), and with higher levels of education (Mikuls et al., 2003) have been previously associated with increased use of NSAIDs (Vina et al., 2021). Among the NHANES 1999–2004 population, Davis et al. (2017) found that former smokers were significantly more likely to use NSAIDs than non-smokers and that this effect was specific to aspirin use. Unfortunately, to the best of our knowledge, there are no previous studies on analyses of the use of NSAIDs and parenthood. Although the results of such studies are not homogeneous, our study found similarities to many of them. Therefore, based on the current study results, educational programs regarding the appropriate use of NSAIDs must preferably be aimed directly at older respondents, smokers, and those with children.

As seen from the regression analyses, behaviors regarding the appropriate use of NSAIDs were significantly higher among respondents with less knowledge (*p*-value <0.000) and more positive attitudes (*p*-value <0.001). As confirmed with previous literature, people’s behaviors are strongly influenced by their level of knowledge as well as attitudes, even if not specifically regarding the use of NSAIDs. This is particularly true with regard to the use of health services and NSAIDs. In the study by Vina et al. (2021), it was reported that appropriate NSAID use behaviors are influenced by knowledge levels as well as attitudes and that there are non-modifiable factors in NSAID use, such as income, health insurance, and quality of social relations (Vina et al., 2021).

The results of the current study show that people who are more aware about NSAIDs use them more inappropriately. Furthermore, the results indicate a strong correlation between correct answers to the questions concerning appropriate use of NSAIDs and younger women with higher levels of education. Therefore, health education programs aimed at using NSAIDs must not only address increasing knowledge but also appropriate use of these drugs.

Therefore, the main objective of this study was to identify specific target populations in a metropolitan area toward whom health education programs aimed at developing correct behaviors about NSAID use could be addressed. Hence, the knowledge, attitudes, and behaviors regarding the appropriate use of NSAIDs were evaluated in a large cosmopolitan cohort. The analyzed population appears to have knowledge gaps on the subject as well as non-excellent attitudes. The results of this study show a statistically significant correlation between attitudes regarding the appropriate use of NSAIDs and less knowledge, indicating that direct knowledge interventions are needed to improve attitudes regarding the appropriate use of NSAIDs. The findings of this study indicate that health programs are needed for populations in metropolitan areas to increase the appropriate use of NSAIDs by focusing specifically on male, older, single, and less-educated individuals through improved knowledge as well as on young women with high levels of education. Health programs should also be implemented more directly to improve people’s behaviors toward the appropriate use of NSAIDs by focusing specifically on older people, smokers, and those with children, in that order. Indeed, behaviors regarding the appropriate use of NSAIDs are statistically significant in younger respondents, non-smokers, and those without children. In conclusion, the inappropriate use of NSAIDs has a strong impact on public health and on the health economy, so alternative solutions should be aimed at promoting additional restrictive regulations on prescriptions and professional consultations before NSAID use.

There are a few limitations to this study that should be taken into account when interpreting the results. A significant constraint of this research is that it depended on self-reported behaviors acquired from questionnaires that were not previously validated. This could have resulted in social desirability bias, aside from the possibility of subjective responses. Nevertheless, by guaranteeing participant anonymity and confidentiality, the study made an effort to address this problem. Furthermore, the limited sample size of the study in comparison to the population of Naples restricts the applicability of the findings. Although the use of a KAP-based questionnaire was effective in assessing the knowledge, attitudes, and practices regarding NSAID use, it might not have captured all the variables influencing the beliefs and actions related to NSAID usage. Another limitation of this work is the lack of detailed monitoring guidelines within the scope of the research, which restricts the ability to provide specific recommendations for healthcare professionals regarding NSAID usage in the studied population. Therefore, despite efforts to ensure data accuracy and reliability, it is important to recognize any potential limitations of this study. Although the sample size was intended as representative of the target population, it is conceivable that it constrains the broader applicability of our conclusions.

## Data Availability

The original contributions presented in the study are included in the article/Supplementary Material, and any further inquiries may be directed to the corresponding author.
